# Relaxin family peptide receptors Rxfp1 and Rxfp2: mapping of the mRNA and protein distribution in the reproductive tract of the male rat

**DOI:** 10.1186/1477-7827-5-29

**Published:** 2007-07-10

**Authors:** Marcelo Filonzi, Laís C Cardoso, Maristela T Pimenta, Daniel BC Queiróz, Maria CW Avellar, Catarina S Porto, Maria FM Lazari

**Affiliations:** 1 Department of Pharmacology, Section of Experimental Endocrinology, Federal University of São Paulo, Rua Três de Maio, 100, CEP 04044-020, São Paulo, SP, Brazil

## Abstract

**Background:**

Relaxin is the endogenous ligand of the G-protein coupled receptor RXFP1, previously known as LGR7. In humans relaxin can also activate, but with lower affinity, the closely related receptor for the insulin-like peptide from Leydig cells, RXFP2, previously known as LGR8. The lack of relaxin impairs male fertility but the precise distribution and the function of relaxin receptors in the male reproductive tract is not known. We investigated the distribution of Rxfp1 and Rxfp2 in the reproductive tract of the male rat and the function of relaxin in the vas deferens, a tissue with high expression of both receptors.

**Methods:**

The presence of mRNA for Rxfp1 and Rxfp2 was investigated in testes, cultured Sertoli cells, epididymis, vas deferens, seminal vesicle, prostate, and spermatozoa by RT-PCR and Southern blot. Protein expression in the testis, vas deferens, primary culture of Sertoli cells, and spermatozoa was assessed by immunohistochemistry and immunofluorescence. The role of relaxin in the vas deferens was evaluated by contractility studies and radioimmunoassay of cAMP production. The effect of relaxin on mRNA levels for metalloproteinase-7 was measured by Northern blot.

**Results:**

Transcripts for Rxfp1 and Rxfp2 were present in almost all parts of the male reproductive tract, with high levels in testis and vas deferens. Both receptors were immunolocalized in late stage germ cells but not in mature spermatozoa, although mRNAs for both receptors were also present in mature spermatozoa. Rxfp1 but not Rxfp2 was detected in cultured Sertoli cells. Strong immunostaining for Rxfp1 and Rxfp2 was seen in muscular and epithelial layers of the vas deferens and in arteriolar walls. Relaxin did not affect contractility and cyclic AMP production of the vas deferens, but increased the levels of mRNA for metalloproteinase-7.

**Conclusion:**

Rxfp1 and Rxfp2 are widely and similarly distributed throughout the male reproductive tract. Our results suggest that Rxfp1 on spermatids and Sertoli cells may be important in spermatogenesis. Relaxin in the vas deferens does not affect contractility, but may affect vascular compliance and collagen and matrix remodeling.

## Background

Relaxin belongs to a superfamily of hormones structurally related to insulin that includes, among others, the relaxins 1, 2 and 3 and the insulin-like peptide from Leydig cells (INSL3) [1]. Humans have three forms of relaxin (H1, H2 and H3), encoded by three different genes, but other mammals like rat, mouse and pig seem to have only two forms of relaxin (relaxins 1 and 3) [1]. The recent discovery that relaxin can bind to and activate the G-protein-coupled receptors (GPCRs) LGR7 and LGR8 [2] was a major breakthrough in the field. Recently the IUPHAR commitee renamed these receptors to RXFP1 and RXFP2 respectively [3]. The human relaxins H1 and H2 and porcine relaxin 1 strongly bind to and activate both RXFP1 and RXFP2 with almost the same affinity, but the rat relaxin 1 binds only weakly to Rxfp2 [2,4]. Relaxin H3 is a selective ligand for RXFP1 but is also able to bind to GPCR135 (RXFP3) and GPCR142 (RXFP4) [5,6]. Insl3 is a selective ligand for Rxfp2 [7].

Relaxin is well known for its relaxing effect on the pubic ligament before parturition, but many other actions have been discovered for this hormone [1,8]. The physiological role of relaxin in the male is not so well defined. In humans and many other mammals, relaxin is produced by the prostate and released almost exclusively in the seminal fluid. The mRNA for RXFP1 and RXFP2 has been detected in testis and in prostate, and the mRNA for RXFP2 has also been detected in gubernaculum [3]. A detailed study of the expression of Rxfp2 in the testis identified the mRNA in Leydig and germ cells of the rat and the protein in human Leydig and germ cells [9]. RXFP2 was also present in the human epididymis [9].

With the recent availability of knockout animals for relaxin or its receptors it has been possible to establish the physiological importance of this hormone. Relaxin has an antifibrotic effect in several tissues, and the relaxin knockout mouse is a model of fibrosis [10-12]. Relaxin interferes with collagen metabolism and increases the expression and activity of metalloproteinases (MMPs) in uterine, cardiac, vascular and renal tissues [13-16]. In the reproductive tract of female mice, the disruption of the relaxin or the *Rxfp1 *gene causes the same abnormalities: an absence of the relaxation and elongation of the interpubic ligament and impaired nipple development [17,18]. In male mice the disruption of the relaxin gene causes a delayed development of the reproductive tract, with an arrest of the sperm maturation [10], but disruption of *Rxfp1 *does not always cause the same abnormalities. Kamat et al. [19] did not find abnormalities in the testes and prostate of *Rxfp1 *knockout mice. Krajnc-Franken et al. [18], using a different strain of knockout mice, observed impaired spermatogenesis, leading to azoospermia and reduced fertility in animals from the first generations but the following generations or older animals had normal fertility.

The interaction of Insl3 with Rxfp2 controls the differentiation of gubernaculum, the caudal genitoinguinal ligament critical for testicular descent, and deletion of *Insl3 *or *Rxfp2 *causes cryptorchidism [20]. Transgenic overexpression of relaxin did not prevent cryptorchidism in *Insl3*-knockout animals [21]. Therefore it seems that relaxin does not physiologically activate RXFP2, although both RXFP1 and RXFP2 can bind relaxin in vitro. Nevertheless, it is possible that binding of relaxin to RXFP2 contributes to some of the actions of relaxin in some species.

The aim of the present study was to map the expression of Rxfp1 and Rxfp2 in the reproductive tract of the male rat. The presence of messenger RNA was investigated in testis, primary culture of Sertoli cells, epididymis (caput and cauda), vas deferens, seminal vesicle, prostate, and spermatozoa by RT-PCR and Southern blot. The localization of both receptors was investigated by immunostaining in the vas deferens, testis, primary culture of Sertoli cells, and spermatozoa. The functional role of relaxin was investigated in the vas deferens by assessing the ability of the hormone to affect contractility, cyclic AMP production, and the transcription of metalloproteinase-7 (Mmp-7), a member of the stromelysin subfamily of MMPs preferentially expressed in epithelial cells of the reproductive tract and present in human semen [22,23].

## Methods

### Animals and biological samples

Male Wistar rats were housed in the Animal Facility at the Instituto Nacional de Farmacologia, UNIFESP-EPM, and maintained on a 12 h light, 12 h dark lighting schedule, at 23°C, with food and water available *ad libitum*. All procedures were approved by the Research Ethical Committee from UNIFESP-EPM.

Tissues were removed from 120-day old rats. Sertoli cells were isolated from testes removed from 15-day old rats and cultured as previously described [24].

To obtain spermatozoa from the epididymis, a small incision was made in the cauda epididymis and a syringe filled with PBS (10 mM sodium phosphate, 0.15 M NaCl, pH 7.4) inserted into the vas deferens. The epididymal content was collected in a tube by retrograde washes. The samples were washed with PBS and centrifuged at 1000 × g for 10 min at room temperature. Somatic contaminants were removed by two sequential centrifugations at 400 × g for 20 min at room temperature through a gradient of 40:80 Percoll (Amersham Biosciences, Piscataway, NJ) in PBS. The pellet containing the viable spermatozoa was washed once with PBS and used for total RNA preparation and immunofluorescence.

### Reverse transcriptase reaction followed by polymerase chain reaction (RT-PCR)

Total RNA was obtained with the TRIzol reagent (Invitrogen, Carlsbad, CA) according to the standard protocol [25] and checked for ribosomal RNA integrity by agarose gel electrophoresis. A 2:1 ratio of sharp and clear ethidium bromide stained 28S:18S ribosomal RNA bands was observed for all samples. The first strand cDNA was synthesized at 42°C with the Thermoscript RT-PCR System (Invitrogen) and the random hexamers supplied with this kit.

The primers designed for amplification of Rxfp1 and Rxfp2 transcripts were complementary to regions in different exons to exclude amplification due to genomic DNA contamination. Table 1 presents a list of all primers used in the present study. The housekeeping gene glyceraldehyde-3-phosphate dehydrogenase (Gapdh) was amplified as a control of the amount of cDNA used in the PCRs. The size of the expected products was compared to a DNA ladder (1 Kb plus or 100 bp ladder, Invitrogen). PCR products were subjected to automated DNA sequencing with the DYEnamic ET Terminator Sequencing Kit (Amersham Biosciences).

**Table 1:** Oligonucleotides used for RT-PCR.

Transcript	Orientation	Sequence	Nucleotide position
Rxfp1 [GenBank:NM_201417]	forward	5'-TGGAGCCCAGATTTATTCAGTGG-3'	1722 to 1744 (exon 14)
	forward	5'-TGTGACGAAGCCAATTTACG-3'	337 to 356 (exon 4)
	reverse	5'-GCCACATTTCCACCCAGATGAATG-3'	2173 to 2150 (exon 16)
Rxfp2 [GenBank:NM_001012475]	forward	5'-TTCAGCGACCTTCACCTTCT-3'	922 to 941 (exon 11)
	reverse	5'-CTTCTGCTTGGTCGTAATGAAGTG-3'	1696 to 1673 (exon 14)
Mmp-7 [GenBank:NM_012864]	forward	5'-CGGAGATGCTCACTTTGACA-3'	603 to 622
	reverse	5'-TGCAAAACCCATCCACAGTA-3'	924 to 905
Gapdh [GenBank:NM_017008.3]			
	forward	5'-TGGGAAGCTGGTCATCAACGG-3'	262 to 282
	reverse	5'-TGGCAGTGATGGCATGGACTG-3'	620 to 600

The conditions for the PCRs were as follows: 2 μL cDNA, 0.5 to 1.0 μM of each primer, 0.2 to 0.6 mM dNTP mixture, 2 or 4 mM MgCl_2_, 2.5 U Taq polymerase (Invitrogen), in a 25 μL final volume. To validate a semiquantitative analysis of the results, we determined the linear range for the PCRs, measuring the amount of product in function of the number of thermal cycles. To amplify the transcript from a transmembrane region of Rxfp1 (exon 14 to exon 16) the following thermal cycles were used: 1 denaturing cycle at 94°C for 5 min; 25 to 45 cycles at 94°C for 1 min, 46°C for 1 min and 72°C for 2 min, with a final extension at 72°C for 7 min. To amplify the transcript from aminoterminal to transmembrane region (exons 4 to 16) of Rxfp1 the following thermal cycles were used: 1 denaturing cycle at 94°C for 5 min; 35–55 cycles at 94°C for 1 min, 55°C for 1 min and 72°C for 2 min, with a final extension at 72°C for 7 min. For amplification of Rxfp2 the following cycle program was used: 1 denaturing cycle at 94°C for 5 min; a touch down of 4 cycles at 94°C for 1 min, 55°C for 1 min and 72°C for 2 min, 4 cycles at 94°C for 1 min, 50°C for 1 min and 72°C for 2 min, followed by 15 to 35 cycles at 94°C for 1 min, 45°C for 1 min and 72°C for 2 min, with a final extension at 72°C for 7 min. For amplification of the Mmp-7 and Gapdh transcripts we used an initial denaturation at 94°C for 5 min, 20 to 40 cycles at 94°C for 1 min, 62°C for 1 min and 72°C for 2 min, with a final extension at 72°C for 7 min. We chose 35 cycles for Rxfp1 (transmembrane region transcript), 50 cycles for Rxfp1 (aminoterminal and transmembrane regions), 25 cycles for RXFP2 and 30 cycles for Gapdh and Mmp-7 to assure the linear range of the exponential curve of PCR amplification. The PCR products were resolved on agarose gels (1.5%) containing ethidium bromide (0.5 μg/ml) and visualized under UV transilumination. The bands corresponding to the expected size were submitted to densitometric analysis with Scion Image software (Scion Corp., 2000) and normalized based on the intensity of the Gapdh related product.

### Southern blot analysis

In tissues with low expression of Rxfp1 and Rxfp2, the RT-PCR products were submitted to a Southern blot analysis. The PCR products were subjected to electrophoresis in a 1.5% agarose gel. The gel was denatured and neutralized and the nucleic acid bands were transferred by capilarity to a nylon membrane (Zeta Probe GT, Bio-Rad, Hercules, CA). Membranes were cross-linked with UV light and prehybridized for 3 h at 65°C in 6 × SSC (standard saline citrate) containing 5 × Denhardt's solution, 0.5% SDS, and 100 μg/mL sheared denatured salmon sperm DNA. Hybridization was performed overnight at 65°C in the same solution containing probes that were labeled with ^32^P (T7 Quick Prime kit, Amersham Biosciences). The Rxfp1 probe was a 452 bp PCR product corresponding to nucleotides 1722 – 2173 of the rat Rxfp1 cDNA. The Rxfp2 probe was a 305 bp product corresponding to positions 1392 to 1696 of the rat Rxfp2 cDNA. The membranes were washed 3 times for 15 min with 2 × SSC/0.1% SDS at room temperature, and 2 times for 15 min with 1 × SSC/0.1% SDS at 65°C. Membranes were exposed for autoradiography and results were analyzed by densitometry with Scion Image software. The size of the ^32^P-labeled PCR products was always compared with the standard DNA ladder and with the product from myometrium. We did not detect any non-specific hybridization with the ^32^P-labeled probes under the high stringency conditions used in the Southern blot.

### Recording of isometric contractions of the uterus and the vas deferens

Porcine relaxin was obtained from National Hormone Peptide Program (Torrance, CA). The vas deferens was removed from 120-day old male rats. As a control we used the uterus of rats that received a subcutaneous injection of 50 μg estradiol hexahydrobenzoate (Benzoginoestryl, Abbott, Brazil) 16 h before the experiments. Tissues were suspended under 0.5 g tension in 10 mL chambers containing aerated nutrient solution [26] (composition mM: NaCl, 137; KCl, 5.6, CaCl_2 _1.8, NaH_2_PO_4 _0.4, NaHCO_3 _15 mM and glucose 8) at 30°C. Isometric contractions were measured by force-displacement transducers connected to a physiograph (Gemini 7070, Ugo Basile, Comerio VA, Italy).

To test if relaxin induces relaxation of smooth muscles we used three protocols. In the first protocol we took advantage of the fact that the vas deferens of castrated animals develops spontaneous contractions [27]. The spontaneous isometric contractions of vas deferens isolated from 30 days castrated rats were recorded for 30 minutes before and after addition of relaxin (0.01, 0.05, 0.1 or 0.5 μg/ml) or forskolin (50 μM; Sigma Chemical Company).

In the second protocol, we tested the contractile response of the organ to 2.5 μM noradrenaline before and after 30 minutes of incubation with relaxin (0.01, 0.05, 0.1 or 0.5 μg/ml) or forskolin (50 μM).

For the third protocol, the vas deferens was pre-contracted by depolarizing the cells with a solution in which Na was replaced with an equimolar amount of K. The resulting response consisted of a phasic contraction that lasted about 1 min, followed by a less intense tonic contraction that lasted about 10 min. Relaxin (cumulative concentration-response curve with 0.01, 0.05, 0.1 and 0.5 μg/ml), forskolin (50 μM), or IBMX (500 μM) were added 5 min after addition of the depolarizing medium, when the tonic contraction was stable. In control experiments the uterus was pre-contracted with a depolarizing solution where 15% of the NaCl was substituted by an equimolar amount of KCl [28]. After stabilization of the contraction, relaxin (0.25 μg/ml) was added.

### Cyclic AMP assay

The vas deferens was removed from 120-day old rats, freed from connective tissue, opened, and cut in squares of approximately 5 mm^2^. The fragments were incubated for 30 min at 30°C in 6 well plates with gentle shaking and aeration in 1 ml nutritive solution [26] containing 0.5 mM of the phosphodiesterase inhibitor isobuthyl-methylxanthine (IBMX, Sigma Chemical Company, St. Louis, MO). Tissues were stimulated for 10 minutes with 0.1, 0.5, 1, 2, or 5 μg/mL of porcine relaxin or with 50 μM forskolin (Calbiochemical, San Diego, CA). Controls were incubated with PBS. The reaction was stopped on ice. The tissue fragments were immediately frozen in liquid nitrogen and homogenized in cold 6% trichloroacetic acid to give a 10% (w/v) homogenate. The homogenate was centrifuged at 2,000 × g for 15 min at 4°C. The supernatant was washed 5 times with 5 volumes of water saturated diethyl ether and the remaining aqueous phase was dried in a speed vac. Cyclic AMP content was measured by radioimmunoassay (cyclic AMP ^125^I Biotrak assay system, Amersham Biosciences). The values were read from a standard curve obtained with 25–1600 fmol cyclic AMP. Results were expressed as a percentage of the value of nonstimulated controls and represent the mean and SEM of three independent experiments.

### Detection of mRNA for metalloproteinase-7 (Mmp-7) in the vas deferens after relaxin treatment

The vas deferens was removed, freed of connective tissues, and cut in four. The pieces were left equilibrating for 30 min at 30°C in 1 ml nutritive solution [26] in 6 well plates with gentle shaking and aeration. The control tissue (no relaxin) was incubated for an additional 120 min in the medium. The other tissues were similarly incubated for 120 min, with relaxin (1 μg/ml) present during the last 30, 60, or 120 min of the incubation period. The reaction was stopped on ice and the tissue fragments were immediately frozen in liquid nitrogen. Total RNA was extracted with TRIzol as described earlier.

Mmp-7 mRNA levels were measured by Northern blot as follows. Five μg total RNA were separated on an agarose/MOPS gel. The separated RNAs were transferred by capilarity to a nylon membrane and crosslinked with UV light. The membranes were prehybridized and hybridized as described for the Southern blot protocol with a ^32^P-labeled 322 bp PCR product corresponding to nucleotides 603 to 924 of the rat Mmp-7 cDNA. The membranes were then washed and exposed for autoradiography and the results were analyzed as described for the Southern blot. Membranes were stripped and rehybridized using a ^32^P labeled probe specific for Gapdh, obtained by PCR as described before. After autoradiography and densitometry, results were normalized based on Gapdh expression.

### Immunohistochemistry

The uterus (control), vas deferens and testis from 120 day old rats were removed and frozen in liquid nitrogen. Sections of the frozen tissues (8 μm) were obtained in cryostat at -20°C and transferred to silanized slides. The sections were kept for 30 minutes at room temperature, fixed for 20 minutes in 4% paraformaldehyde buffered with PBS, incubated for 5 minutes in 0.1 M glycine, and treated with 0.3% hydrogen peroxide for 10 minutes to remove endogenous peroxidase activity. The sections were incubated in blocking buffer (PBS with 0.01% saponin and 3% albumin) for one hour at room temperature in a humidified chamber. Endogenous biotin was blocked with the Avidin/Biotin Blocking Kit (Vector Laboratories, Burlingame, CA).

The sections were incubated overnight at 4°C with the primary antibodies diluted in blocking buffer (1:100, 1:200 or 1:300). As a primary antibody against Rxfp1 we used rabbit anti-human LGR7 (735–757) (H001-53, Phoenix Pharmaceuticals, Belmont, CA). The primary antibody against Rxfp2 was rabbit anti-human LGR8 (737–754) (H001-54, Phoenix Pharmaceuticals). Both antibodies recognize rat proteins and have been well characterized for human [29,30] and rat tissues [31,32]. The sections were rinsed 3 times, 5 min each, with the blocking buffer, and incubated for 1 h with the secondary antibody (1:300 dilution of biotinylated goat anti-rabbit IgG (Santa Cruz Biotechnologies, Santa Cruz, CA). The sections were rinsed again 3 times with blocking buffer and incubated for 1.5 h at room temperature with avidin-conjugated horseradish peroxidase complex (ABC system, Santa Cruz Biotechnologies). Peroxidase activity was revealed by incubation with 3,3'-diaminobenzidine (DAB, 0.5 mg/ml) in 0.01% H_2_O_2 _for 3 min. The sections were washed, dried at room temperature, and counterstained with methylene blue.

Slides were examined with a Nikon E800 microscope (Melville, NY) and images were captured with a digital camera and analyzed with Media Cybernetics software (Silver Spring, MD). Negative controls (tissues incubated with non-immune rabbit serum instead of the primary antibody) were run in parallel with the tissues of interest.

### Immunofluorescence

Sertoli cells were plated on gelatin-coated coverslips in 6-well plates (2 × 10^5 ^cells/well). After five days in culture they were fixed with 3.7% formaldehyde for 30 min at 37°C. They were washed 3 times with PBS and blocked with PBS containing 3% bovine serum albumin (BSA) for 30 min at room temperature. They were washed again with PBS. The cells were incubated for 2 h at room temperature with primary antibody (1:100 or 1:300 dilution of rabbit anti-human RXFP1 or RXFP2). To permeabilize samples, 0.1% saponin was used in the blocking buffer and during incubation with the antibodies. Cells were washed and incubated with 1:300 dilution of a fluorescent secondary antibody (30 min, goat anti-rabbit IgG, Alexafluor 594, Molecular Probes, Eugene, OR).

Viable spermatozoa were separated with a Percoll gradient as described before, spread on silanized slides, and dried for 30 min. They were fixed with 2% formaldehyde, incubated for 5 min with PBS containing 0.1 M glycine, rinsed with PBS, and incubated with blocking buffer (PBS with 1% BSA and 0.1% saponin) for 10 min. Cells were washed with PBS and incubated for 1 h with a 1:50, 1:100, or 1:300 dilution of the primary antibody. They were washed again and incubated for 30 min with the secondary antibody. Nuclei were visualized by staining with DAPI (4', 6-diamine-2-phenylindol). Negative controls were run in the absence of the primary antibody. The slides were examined with a Nikon E800 fluorescence microscope.

### Statistical analysis

Data were expressed as mean ± SEM. Statistical analysis was carried out by analysis of variance (ANOVA) followed by Newman-Keuls test for multiple comparisons or by Student's *t*-test to compare the differences between two data (Sigmastat version 2.03, SPSS Inc). *P *values < 0.05 were accepted as significant.

## Results

### Rxfp1 and Rxfp2 messenger RNA in the reproductive tract of the male rat

**Figure 1:**
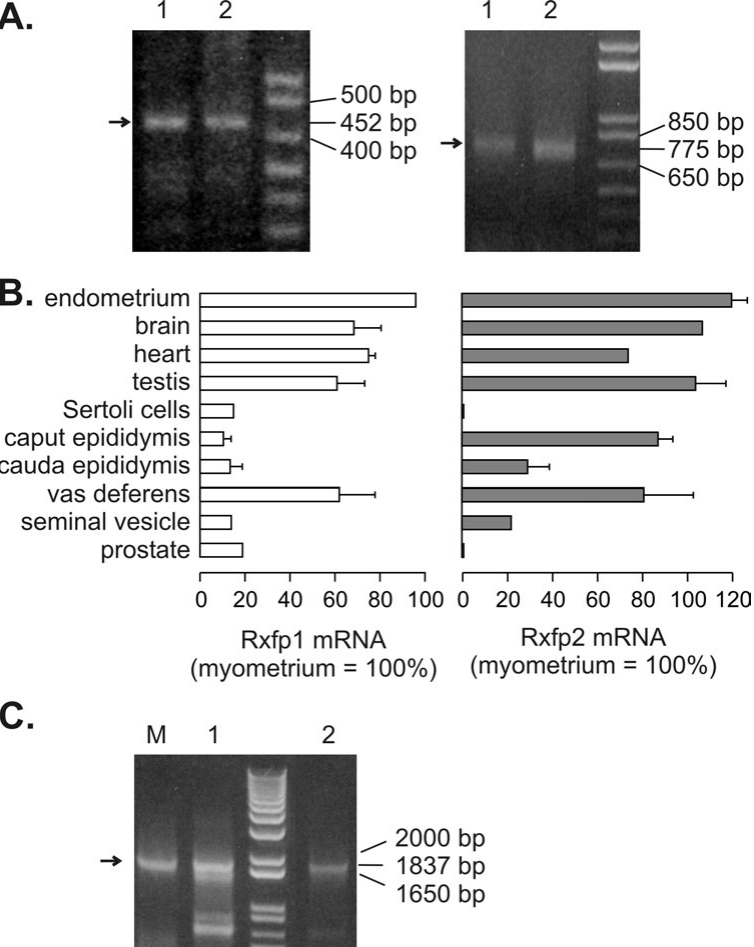
Rxfp1 and Rxfp2 transcripts are present throughout the reproductive tract of the male rat. [A] Agarose gel electrophoresis with Rxfp1 (left panel, 452 bp product) and Rxfp2 (right panel, 775 bp product) transcripts amplified by RT-PCR from the testis (lane 1) and vas deferens (lane 2). Arrows indicate the expected product. [B] Densitometric analysis of Southern blots of Rxfp1 (left) and Rxfp2 (right) transcripts amplified by RT-PCR from various tissues of the male reproductive tract. Results are mean ± SEM of measurements with RNAs from 3 – 5 independent experiments, each with a different animal. [C] RT-PCR analysis of the presence of exon 4 in Rxfp1 transcripts (1837 bp product) in myometrium (lane M), testis (lane 1) and vas deferens (lane 2). Result is representative of 3 independent experiments, each with a different animal.

In the testis and the vas deferens transcripts for Rxfp1 (452 bp product) and Rxfp2 (775 bp product) could always be easily visualized on ethidium bromide-stained agarose gels (Figure 1A and 1C). A Southern blot analysis after RT-PCR with several rat tissues allowed mapping the presence of the mRNA for Rxfp1 and Rxfp2 even in tissues of the male reproductive tract with low expression of the transcripts (Figure 1B). Our aim was not to quantify the transcripts for both proteins, but these semi-quantitative methods clearly show that mRNA levels for Rxfp1 were high in testis, vas deferens, myometrium, endometrium, heart, and brain, while lower levels of Rxfp1 were found in the other parts of the reproductive tract. The distribution of Rxfp2 mostly paralleled that of Rxfp1. Notable differences were remarkably high Rxfp2 levels in the caput epididymis, and the absence of Rxfp2 in the prostate.

Since a splice variant for RXFP1 was detected in rat tissues with a deletion in exon 4 that leads to a truncated form the protein [33], we investigated whether RXFP1 transcripts detected in testis and vas deferens contained that region, using a forward primer complementary to a region in exon 4. Figure 1C shows that a transcript containing exon 4 to exon 16 (1837 bp product) was present in the myometrium, testis and vas deferens.

**Figure 2:**
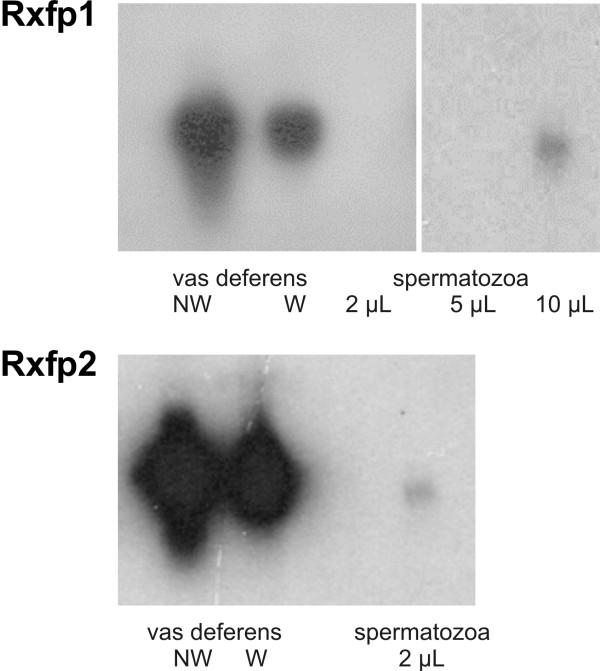
Autoradiograms of the Southern blot analysis of the presence of Rxfp1 and Rxfp2 mRNA in the vas deferens (NW = not washed, W = washed to remove spermatozoa) and in spermatozoa removed from the cauda epididymis. The amount of cDNA used from the vas deferens was 2 μL. Exposure to film was 2 h for Rxfp1 and 1 h for Rxfp2 transcripts. Results are representative of 3 independent determinations, each one with a different animal.

We detected a weak signal for both Rxfp1 and Rxfp2 transcripts in spermatozoa removed from the cauda epididymis (Fig. 2), but we had to use larger amounts of cDNA to visualize the signal for the Rxfp1 transcript. The high levels of Rxfp1 and Rxfp2 mRNA in the vas deferens were not due to spermatozoa, since the mRNA levels in the vas deferens were about the same after washout of the luminal content.

### Immunolocalization of Rxfp1 and Rxfp2 proteins in vas deferens and testis

**Figure 3:**
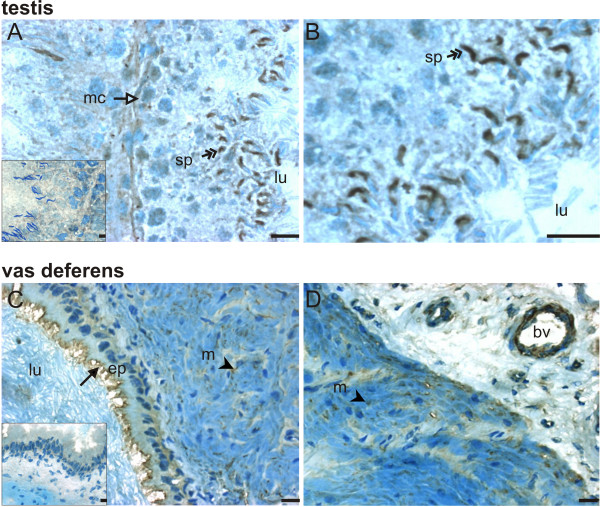
Localization of Rxfp1 protein in the testis (A, B) and vas deferens (C, D). Immunohistochemistry for Rxfp1 (1:100 dilution of primary antibody) was done in cryosections of the testis and vas deferens; sections were counterstained with methylene blue. Inserts show controls incubated with non-immune rabbit serum instead of primary antibody. Scale bars are 5 μm. Results are representative of at least 3 independent determinations, each one with a different animal. bv = blood vessel, ep = epithelial layer, lu = lumen, m = muscular layer, mc = myoid cells, sp = spermatid. Arrows indicate labeled epithelial cells, arrow heads indicate labeled muscular cells, double-headed arrows indicate labeled spermatids and open-headed arrow indicates labeled myoid cells.

**Figure 4:**
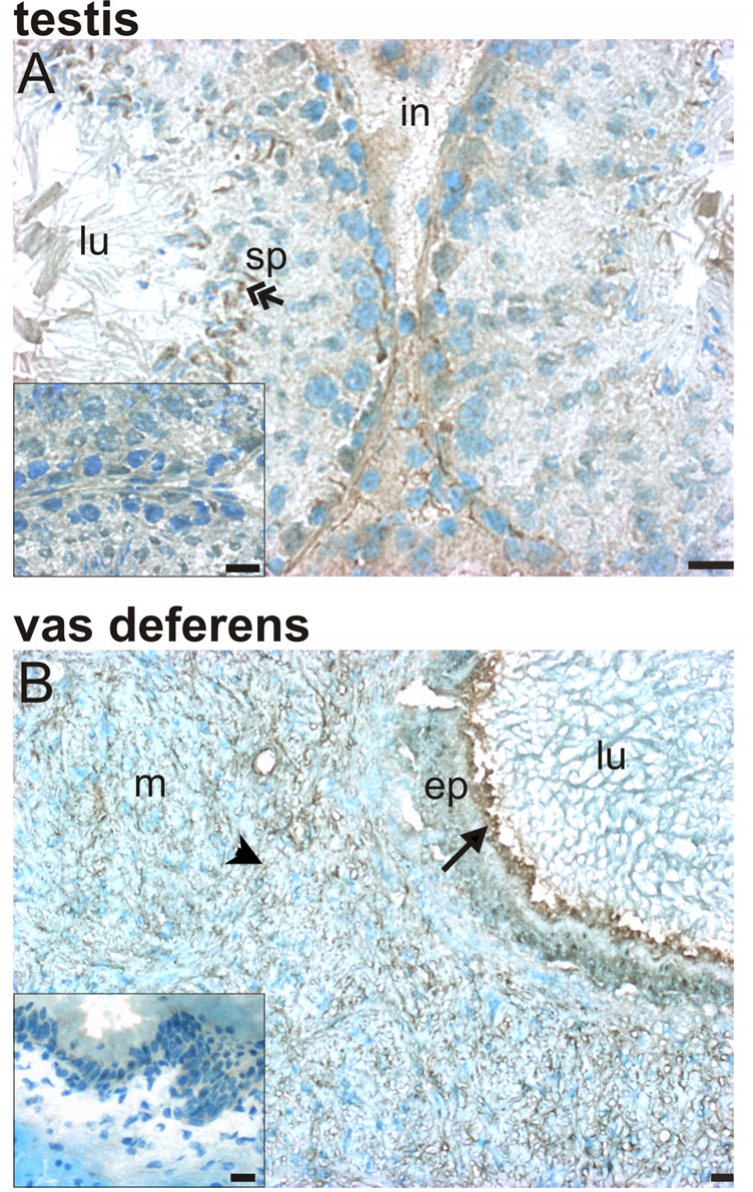
Localization of Rxfp2 protein in the testis (A) and vas deferens (B). Immunohistochemistry for Rxfp1 (1:100 dilution of primary antibody) was done in cryosections of the testis and vas deferens; sections were counterstained with methylene blue. Inserts show controls incubated with non-immune rabbit serum instead of primary antibody. Scale bars are 5 μm. Results are representative of at least 3 independent determinations, each with a different animal. ep = epithelial layer, in = interstitium, lu = lumen, m = muscular layer, sp = spermatid. Arrows indicate labeled epithelial cells, arrow heads indicate labeled muscular cells, and double-headed arrows indicate labeled spermatids.

We performed immunohistochemistry to examine what cell types account for the high levels of mRNA for Rxfp1 (Fig. 3) and Rxfp2 (Fig. 4) in the vas deferens and the testis. Intense Rxfp1 immunostaining was detected in late stage germ cells from the testis, but not in early stage germ cells (Figure 3A and 3B). Diffuse staining was detected in the basal compartment of the tubuli and in myoid cells (Figure 3A). No significant immunostaining was seen in negative controls incubated with non-immune serum instead of primary antibody (inserts). Strong immunostaining for Rxfp1 was detected in the apical portion of epithelial cells and in the longitudinal and circular muscular layers of the vas deferens (Fig. 3C). We also detected Rxfp1 in the wall of blood vessels. Fig 3D shows an example of Rxfp1 staining in what appears to be an arteriole, based on vessel diameter and wall thickness, with most of the staining localized in putative smooth muscle.

Rxfp2 expression similarly was high in late stage germ cells and absent in early stage cells, but diffuse Rxfp2 immunostaining was also present in the interstitial compartment (Figure 4A). It is interesting to note that the immunostaining for both Rxfp1 (Fig. 3A) and Rxfp2 (Fig. 4A) in adjacent tubuli varied, suggesting that the expression of these proteins may be specific for certain stages of spermatogenesis. The pattern of expression of Rxfp2 in the vas deferens was similar to that of Rxfp1: staining was detected in the apical portion of epithelial cells and in the muscular layers of the organ (Figure 4B).

**Figure 5:**
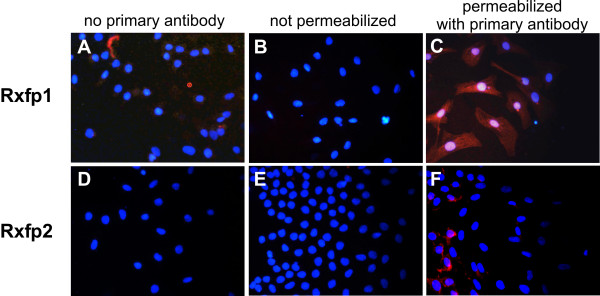
Rxfp1 and Rxfp2 receptors in cultured Sertoli cells. Receptors were labeled red with immunofluorescent antibody against intracellular domains of the receptors, nuclei were labeled blue with DAPI. A and D: assay with permeabilized cells in the absence of the primary antibody. B and E: assay with non-permeabilized cells in the presence of the primary antibody. C and F: assay with permeabilized cells in the presence of the primary antibody. Rxfp1 antibody dilution 1:300, Rxfp2 antibody 1:100. Results are representative of 3 different cell cultures.

Spermatozoa collected from the cauda epididymis did not show any immunofluorescence indicative of Rxfp1 and Rxfp2 receptors (data not shown). Primary cultures of Sertoli cells presented immunofluorescence for Rxfp1 (Figure 5C), but not for Rxfp2 (Figure 5F). No immunofluorescence for Rxfp1 was detected in Sertoli cells incubated without primary antibody (Figure 5A) or with non-immune serum instead of primary antibody (data not shown), and in unpermeabilized Sertoli cells where the primary antibody did not have access to the epitope in the carboxyterminal region of the receptor (Figure 5B).

### Relaxin fails to affect contractile activity of the vas deferens

**Figure 6:**
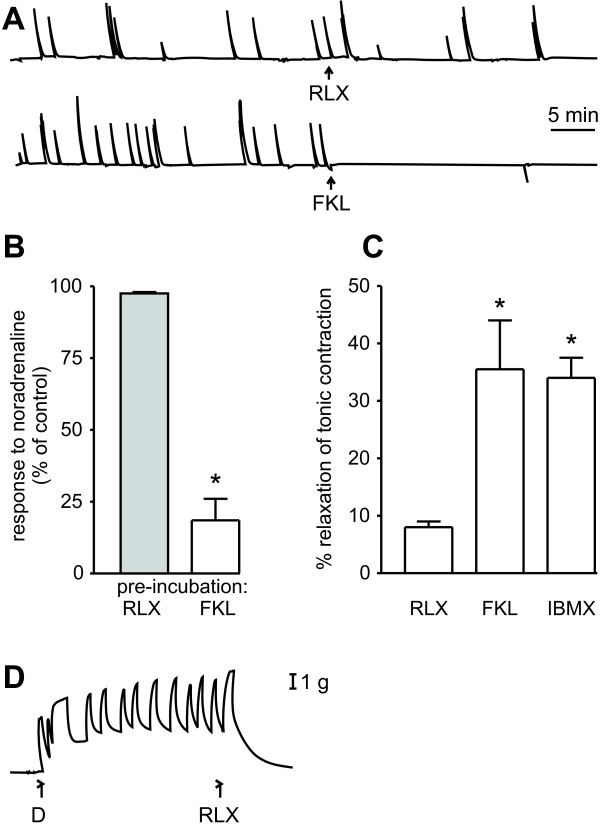
Relaxin fails to affect contractility of the vas deferens. [A] Effects of relaxin (RLX, 0.5 μg/ml) and forskolin (FKL, 50 μM) on spontaneous contractions of the vas deferens from castrated rats. [B] Effect of pre-treatment for 30 min with RLX (0.5 μg/ml) or FKL (50 μM) on contractions of the vas deferens induced by 2.5 μM noradrenaline; * *P *< 0.05 compared to relaxin (Student's *t *test). [C] Effect of RLX (0.5 μg/ml), FKL (50 μM) and IBMX (500 μM) on contractions of the vas deferens induced by incubation in a depolarizing solution; *P < 0.05 compared to relaxin (Newman Keuls). [D] Control experiment showing the relaxing effect of relaxin (RLX 0.25 μg/ml) in rat uterus. All experimental protocols were repeated at least 3 times with different animals.

To test if the relaxin receptors in muscular layer of the vas deferens were involved in contraction we measured the effect of relaxin on the contractility of the isolated vas deferens. Relaxin (0.01, 0.05, 0.1, and 0.5 μg/mL) failed to affect the spontaneous contractions that are seen in the isolated vas deferens of castrated rats (Fig. 6A; 0.5 μg/mL). Forskolin completely inhibited these contractions. Pre-treatment of the vas deferens with 0.01, 0.05, 0.1 or 0.5 μg/mL relaxin for 30 min did not affect the contractile response to noradrenaline (Fig. 6B; 0.5 μg/mL). This contractile response was strongly reduced by pre-treatment with forskolin. When the vas deferens was precontracted with a depolarizing solution in which Na^+ ^was replaced with K^+^, the contraction was blunted by forskolin (50 μM) and by IBMX (500 μM), but little change was seen after a cumulative dose-response curve with relaxin (a reduction of 8% with a final concentration of 0.5 μg/mL, Fig. 6C). On the other hand, 0.25 μg/mL relaxin completely relaxed the pre-contracted uterus (Figure 6D).

### Relaxin fails to affect the production of cyclic AMP, but increases mRNA levels for MMP-7 in the vas deferens

**Figure 7:**
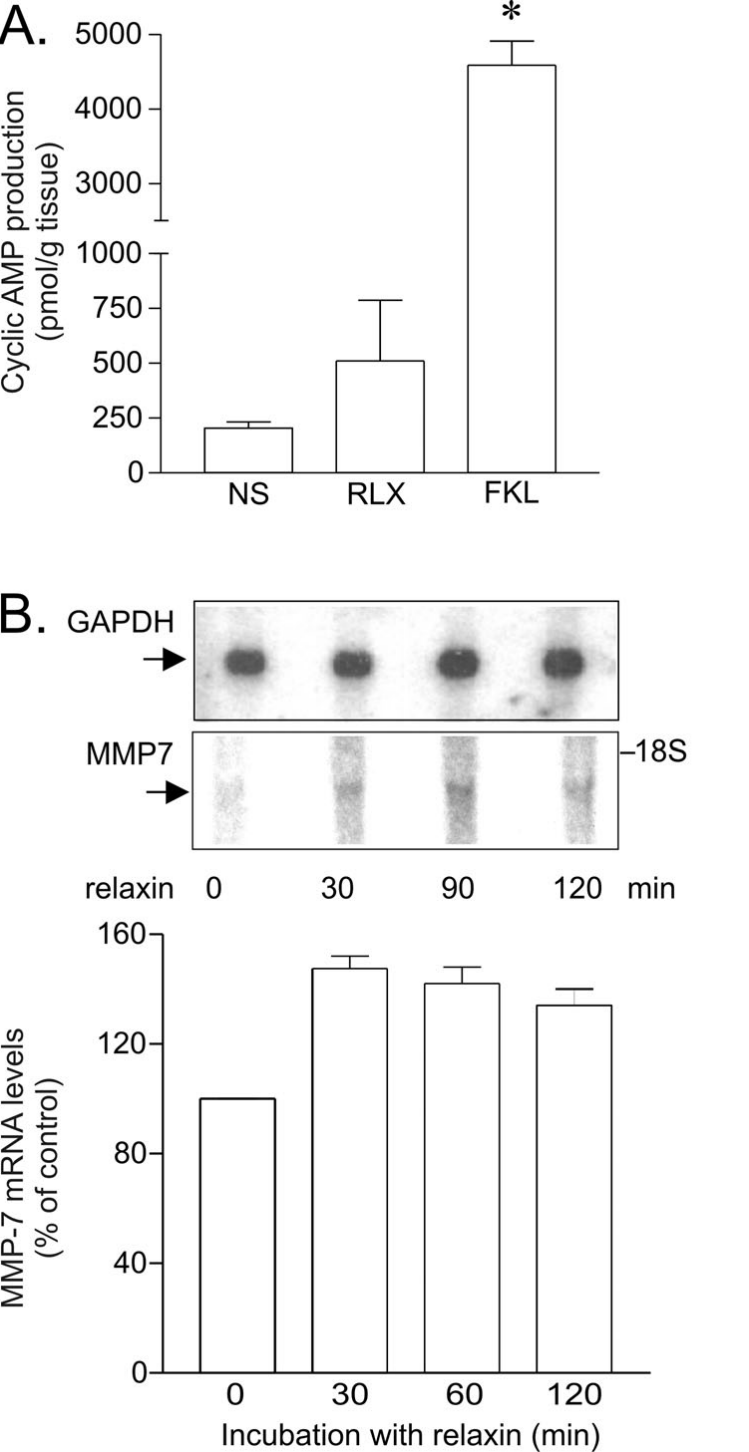
[A] Relaxin fails to affect cyclic AMP production in the vas deferens. Slices of the vas deferens were incubated for 10 min with PBS (control), RLX (5 μg/ml) or FKL (50 μM) and the cyclic AMP production was measured. Results are mean ± SEM of 3 independent experiments. * *P *< 0.05 compared to control (Student's t test). [B] Northern blot analysis of the effect of relaxin on Mmp-7 mRNA levels in the vas deferens. Upper panel shows a representative autoradiogram with Mmp-7 and Gapdh mRNA after various periods of incubation with relaxin (1 μg/mL). Bottom panel shows densitometric analysis of two independent experiments. Error bars indicate SEM.

Since muscular activity in the vas deferens was reduced by drugs that increase cyclic AMP levels, we investigated the ability of relaxin to affect cyclic AMP production in the vas deferens. We found that low to moderate concentrations of relaxin (0.1 to 0.5 μg/ml) did not increase cyclic AMP production (data not shown). High concentrations of relaxin (5 μg/ml) seemed to induce a slight increase in the cyclic AMP production, but this effect was not statistically significant and much smaller than the effect of forskolin (Fig. 7A).

Relaxin is a well known regulator of metalloproteinases expression and activity both in non-reproductive and reproductive tissues. Metalloproteinase 7 (MMP7) is an enzyme that is present in human semen and is expressed particularly in reproductive tissues [34,22,23]. It degrades a wide range of extracellular matrix proteins, including collagen [35], and activates other MMPs, including MMP2 and MMP9, which play a major role in degradation of collagen, and MMP1 and MMP8 [36]. Incubation with 1 μg/ml relaxin, a concentration that did not affect cyclic AMP production, increased the mRNA levels for Mmp-7 in the vas deferens by about 40%, and this increase was maintained with longer periods of incubation (Figure 7B).

## Discussion

We investigated the distribution of the Rxfp1 and Rxfp2 receptors in the reproductive tract of the male rat. These two receptors interact with members of the superfamily of insulin-related hormones, relaxin and Insl3. In human males, relaxin is produced mainly in the prostate [37,38] and released primarily if not exclusively into the seminal fluid [1], where its concentration ranges 1 to 73 ng/mL [39,40]. On the other hand, in males INSL3 is a major secretory product of the testicular Leydig cels [41,42] and is now recognized as a major circulating hormone. In several species, relaxin can activate both Rxfp1 and Rxfp2, and it is still unknown whether the concomitant presence of both receptors may contribute to the final response to relaxin.

Our data show that mRNA for Rxfp1 is present in all tested tissues. High levels were found in the testis and vas deferens, and lower levels in the prostate, Sertoli cells, caput and cauda epididymis, seminal vesicle, and spermatozoa. Immunohistochemistry of the testis showed that Rxfp1 protein is especially abundant in late stage germ cells (elongate spermatids). The location of these receptors on late stage germ cells and on Sertoli cells suggests a role in spermatogenesis. However, while disruption of the relaxin gene is known to cause an arrest in sperm maturation [10], disruption of the *Rxfp1 *gene does not have the same effect [18,19].

Immunohistochemistry of the vas deferens showed receptors in longitudinal and circular muscle layers, in the epithelial layer, and in arterioles. It has previously been shown by immunohistochemistry that relaxin is present in the vas deferens [43]. This relatively neglected part of the male reproductive tract has a complex epithelium with absorptive and secretory functions [44]. Our results suggest that muscular Rxfp1 receptors may be important in the organization of the extracellular matrix, but they do not seem to be involved in acute modulation of contractility. Whether Rxfp1 receptors on the epithelium are involved in regulation of secretion is not yet clear. The presence of Rxfp1 in arterioles suggests that relaxin may regulate local vascular resistance. Both relaxin and Rxfp1 were previously detected in the thoracic aorta and in small renal and mesenteric arteries [45,46], suggesting a more general role of the relaxin-Rxfp1 receptor system in the regulation of local arterial function.

Although Rxfp1 mRNA was present in mature spermatozoa, the levels were low, and Rxfp1 protein was undetectable. Nevertheless, the presence of Rxfp1 receptor on spermatozoa would help explain many studies that suggest that relaxin stimulates sperm motility. For example, antiserum against relaxin reduces sperm motility [47], relaxin stimulates sperm motility and attenuates the decline in the percentage of motile spermatozoa [48], helps restore decreased sperm motility [48], and improves penetration of spermatozoa into cervical mucus [50]. Finally, Carrell et al. [50] demonstrated that recombinant relaxin binds to sperm with high affinity. In contrast, Jockenhovel et al. [52] and Newinger et al. [53] failed to find an effect of relaxin on sperm function. In conclusion, it is not yet clear if spermatozoa contain functional relaxin binding sites, and if they do, whether they are alternative binding sites.

The distribution of Rxfp2 in the testis and epididymis was recently analyzed by Anand-Ivell et al. [9]. Their finding of high Rxfp2 levels in late stage germ cells, the presence of RXFP2 in the epididymis, and the absence of Rxfp2 in Sertoli cells is firmly supported by our results. In addition, we found high levels of Rxfp2 in the vas deferens. Rxfp2 transcripts were also present in the seminal vesicle. The absence of Rxfp2 transcripts in the prostate of the rat contrasts with a study by Klonisch et al. [54] that reported RXFP2 transcripts in epithelial cells of the human prostate.

Our results also show that the distribution of Rxfp1 and Rxfp2 is similar in many tissues and both receptors are expressed in late-stage germ cells. Whether their function is similar remains to be seen. Disruption of the *Rxfp2 *gene causes cryptorchidism, which affects spermatogenesis [20]. However, it seems that Rxfp2 is not essential for spermatogenesis, because if the cryptorchidism that results from the lack of Rxfp2 is surgically corrected at birth, sperm maturation appears normal [20].

Although Rxfp1 receptors were found in the muscular layer of the vas deferens, and although relaxin is known to reduce uterine tone [55], our results clearly suggest that relaxin is not involved in modulation of contractility in the vas deferens. Relaxin did not affect spontaneous contractions of the rat vas deferens, did not relax the depolarized contracted organ, and did not affect the contractile response to noradrenaline, but all these responses were blocked in control experiments with drugs that affect cyclic AMP levels such as forskolin or IBMX.

Relaxin can increase cyclic AMP after interacting with recombinant RXFP1 and RXFP2 in heterologous systems [2,5,56]. Relaxin can stimulate endogenous receptors to increase cyclic AMP in many reproductive [55-57,59] and non-reproductive tissues [3], and other downstream signaling reactions can be activated after production of cyclic AMP [3]. However, our data clearly indicate that relaxin does not stimulate the production of cAMP in the vas deferens. This result is in accordance with previous observations [55]. In rat atria and in fibroblasts from the human lower uterine segment, relaxin also produced little or no cyclic AMP [60,61]. It remains to be determined whether other signaling pathways such as the tyrosine kinase signaling pathway mediate relaxin actions in the vas deferens.

It should be pointed out that activation of RXFP2 by INSL3 reduces cyclic AMP in testicular germ cells and oocytes, and this effect is prevented by treatment with pertussis toxin [62]. Stimulation of the recombinant RXFP2 receptor with INSL3 resulted in a delayed phase coupled to G_0B _[56]. Therefore we speculate that the joint presence of both Rxfp1 and Rxfp2 in the same tissue can result in a more complex regulation of the cyclic AMP pathway. An additional complication is the presence of RXFP1 splice variants, which are found in man [63], rats, and mice [33]. The absence of exon 4 leads to the production of a truncated RXFP1 receptor that can act as an inhibitor of RXFP1 signaling. The Rxfp1 transcripts detected in the present study in the testis and the vas deferens however did contain exon 4. Splice variants for RXFP2 have also been reported [64]. In the rat, the Rxfp2 transcripts found so far in the testis, germ cells and epididymis encoded the full-length receptor, but the gubernaculum produces a Rxfp2 product that lacks exon 3 [9].

Relaxin is well known for its antifibrotic action and for its stimulation of the expression and activity of metalloproteinases. Metalloproteinases are a family of extracellular proteases that interfere with the matrix remodeling by their ability to degrade extracellular matrix components including collagen. Relaxin increases the expression of MMP-2, MMP-9 and MMP-3 in uterine and other tissues [65,65,66,15]. Relaxin has also been shown to enhance in-vitro invasiveness of breast cancer cells, where it increases the production of several metalloproteinases, including MMP-7 [67]. In turn MMP-7 activates MMP-1, -2 -8 and -9 [36]. The majority of the metalloproteinases are produced by mesenchymal cells in the stroma, whereas MMP-7 expression is restricted to epithelium, particularly glandular epithelium [34,22]. Messenger RNA for MMP-7 has been found in epithelial cells lining the efferent duct and in prostatic epithelial cells in mouse and human, and MMP-7 activity has also been detected in human semen [23]. This expression pattern of MMP-7 almost exclusively to reproductive tissues suggests a role in the reproductive function. We here show that MMP-7 mRNA is also present in the vas deferens and is increased by relaxin, suggesting a role for relaxin with regulation of extracellular matrix components, including other MMPs activation, collagen remodeling and apoptosis, as already shown in other tissues. A significant increase in the collagen expression in the prostate and testis of relaxin knockout mice has been documented before [10].

## Conclusion

In this study we detected by RT-PCR and Southern blot the presence of transcripts for the GPCRs Rxfp1 and Rxfp2, both of which are present throughout the male reproductive tract. Rxfp1 on spermatids and Sertoli cells may be important in spermatogenesis. Relaxin in the vas deferens is not involved with contractility, but is probably involved with vascular compliance and collagen and matrix remodeling and/or apoptosis.

## Competing interests

The author(s) declare that they have no competing interests.

## Authors' contributions

MF and LCC contributed equaly for this paper: MF carried out the RT-PCR, Southern blot and immunohistochemistry studies; LCC participated in the RT-PCR and Southern blot studies and carried out the functional assays (cyclic AMP and contractility) and the Northern blot analysis. MTP carried out the immunofluorescence and helped with the cyclic AMP assays. DBCQ helped with the immunohistochemistry studies. MCWA and CSP participated in the design and coordination of the study and helped to draft the manuscript. All authors read and approved the final manuscript.
